# Fully analogue photonic reservoir computer

**DOI:** 10.1038/srep22381

**Published:** 2016-03-03

**Authors:** François Duport, Anteo Smerieri, Akram Akrout, Marc Haelterman, Serge Massar

**Affiliations:** 1 OPERA-Photonique, CP 194/5, Université Libre de Bruxelles (U.L.B.), Avenue Adolphe Buyl 87, 1050 Brussels, Belgium; 2 Laboratoire d’Information Quantique, CP 224, Université Libre de Bruxelles (U.L.B.), Boulevard du Triomphe, 1050 Brussels, Belgium

## Abstract

Introduced a decade ago, reservoir computing is an efficient approach for signal processing. State of the art capabilities have already been demonstrated with both computer simulations and physical implementations. If photonic reservoir computing appears to be promising a solution for ultrafast nontrivial computing, all the implementations presented up to now require digital pre or post processing, which prevents them from exploiting their full potential, in particular in terms of processing speed. We address here the possibility to get rid simultaneously of both digital pre and post processing. The standalone fully analogue reservoir computer resulting from our endeavour is compared to previous experiments and only exhibits rather limited degradation of performances. Our experiment constitutes a proof of concept for standalone physical reservoir computers.

Reservoir computing is a bio-inspired approach for processing time dependent information^1^,^2^,^3^,^4^,^5^. A reservoir computer can be decomposed into three parts, see Fig. 1. The “input layer” couples the input signal into a non-linear dynamical system that constitutes the “reservoir layer”. The internal variables of the dynamical system, also called “reservoir states”, provide a nonlinear mapping of the input into a high dimensional space. Finally the time-dependent output of the reservoir is computed in the “output layer” as a linear combination of the internal variables. The readout weights used to compute this linear combination are optimized so as to minimize the mean square error between the target and the output signal, leading to a simple and easy training process. On the other hand, the values of the internal coupling weights within the input layer and within the reservoir layer are not critical, and can be chosen at random up to some global parameters that are tuned to get the best performance.

One of the key advantages of reservoir computers is that, because only the output layer is trained, training algorithms are efficient and rapidly converge to the global optimum. This simplicity enables reservoir computers to solve a large range of complex tasks on time dependent signals, such as speech recognition^6^, nonlinear channel equalization^3^,^7^,^8^, detection of epileptic seizures^9^, robot control^10^, time series prediction^1^,^3^, financial forecasting, handwriting recognition, etc…, often with state of the art performance. We refer to^11^,^12^,^13^ for recent reviews.

This simplicity and flexibility has also allowed for a breakthrough in analogue information processing, and in particular in optical information processing. The experimental implementations^14^,^15^,^16^,^17^,^18^,^19^,^20^,^21^,^22^,^23^,^24^,^25^,^26^,^27^,^28^,^29^,^30^ of reservoir computing (most of them optical) often report error rates comparable to the best digital algorithms. Most of these experiments, and in particular those that have been able to tackle the most complex tasks, are based on an architecture, introduced experimentally in^15^ (see also the earlier report^31^ and the theoretical proposal^32^,^33^), consisting of a single nonlinear node and a delay line in which the reservoir states are time multiplexed. These experimental demonstrations are further complemented by extensive studies in simulation of alternative or improved optical implementations^34^,^35^,^36^,^37^,^38^,^39^,^40^,^41^.

Despite this intensive research, the potential of reservoir computing in terms of processing easiness and speed has not yet been fully considered. In particular, all previous experiments required either digital pre-processing of the inputs, or digital post-processing of the outputs, or both (i.e. at least either the input layer or the output layer were digitally implemented). This is indeed a major limitation if one intends to use physical reservoir computers as versatile and efficient standalone solutions. Moreover, besides the advantages of speed and versatility, a fully analogue device would allow for the feedback of the output of the reservoir into the reservoir itself, enabling new training techniques^42^ as well as the exploitation of reservoir computers to new kinds of tasks, such as pattern generation^3^,^43^.

Note that some steps towards a fully analogue reservoir have already been taken. In our unpublished manuscript^44^ we showed how to implement an analogue input layer. In fact an analogue input layer is comparatively simpler to implement, as it consists of multiplying the input signal with random weights. The exact values of these weights are not very important, as they can be chosen at random up to some global scaling. Optimisation of the input weights has been considered in^22^,^28^,^40^.

The first report of a reservoir computer with an analogue output layer was given in^20^. This solution was tested on a single task, and the results obtained were not as good as those obtained using a digital output. The difficulty of constructing an analogue output layer is due to the nature of the computation that needs to be carried out. Indeed the output of the reservoir computer is a linear combination, with positive and negative weights, of many internal states, which requires a very high computation accuracy. While this accuracy is obtained naturally with a digital computer, it is rather challenging to reach it with an analogue integrating device.

In the present work we report the first fully analogue reservoir computer. Our implementation takes as input an analogue optical signal, and produces as output an analogue electrical signal proportional to the output requested by the task. We thereby prove that the concept of reservoir computing can be entirely implemented by means of analogue signals handled by analogue components. This opens up the route to new promising developments based on high-bandwidth standalone reservoirs as well as feedback loop reservoirs.

In what follows we first introduce the concept of reservoir computer, and then review the optoelectronic reservoir exploited in the present work. This reservoir, based on a single nonlinear node and a fibre delay loop, was introduced in^17^,^18^. In Sec. 4 we describe the analogue input layer that we implemented in the present work on the basis of our previous study^44^. The input mask (i.e. the weights that determine the coupling between the input and the reservoir states) is given by the sum of two sine functions which could be easily generated by oscillators and should be therefore more suitable for future integration. The analogue output layer discussed in Sec. 5 is an improved version of the solution developed in^20^. In Sec. 6 we study the performance of the fully autonomous reservoir computer on three tasks that are traditionally considered in the reservoir computing community, namely NARMA10, nonlinear channel equalization and radar signal forecasting. Finally we discuss the implications of our work for the future development of photonic reservoir computing.

## Reservoir Computing Basics

A reservoir computer, see^11^,^12^,^13^ for general presentations, is composed of three layers: an input layer, the reservoir itself and an output layer. The input signal is a time series *u*(*n*) where *n*∈ 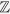 is the discrete time. The internal variables of the reservoir, also called reservoir states, are denoted *x*_*i*_(*n*), *i = 1,…,N* with *N* the number of internal variables. The reservoir is a nonlinear dynamical system that recurrently couples the internal states to each other. The input layer distributes the input *u*(*n*) to the reservoir states with coupling coefficients that vary for each internal state. These coefficients, also called the input mask, are often drawn from a random distribution. The fact that the input mask coefficients vary enriches the dynamics of the reservoir by breaking the symmetry that would occur if the same image of the input would be distributed to all the internal variables. The output layer linearly combines the internal variables *x*_*i*_(*n*) to construct the output *y*(*n*) of the system. Whereas in traditional recurrent neural networks, all the layers are optimised, the reservoir computing technique proposes to only optimise the output layer while the input layer and interconnections within the reservoir are fixed. The optimization of the output layer consists in a linear regression, which greatly simplifies the training of reservoir computers. It is thus possible to use large reservoirs and obtain processing capabilities as good as with other machine learning techniques.

The dynamics (in discrete time) of the internal variables are described by the evolution equation


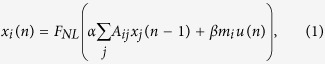


where the matrix *A*_*ij*_ represents the interconnections between the internal variables *x*_*i*_. This matrix is normalized so that its largest eigenvalue is equal to one. The vector *m*_*i*_ represents the input mask. The two parameters *α* and *β* are used to adjust the strength of the feedback signals inside the reservoir and the strength of signals injected into the reservoir. In digital implementations, the nonlinear function *F*_*NL*_ is often taken to be a hyperbolic tangent, but many other nonlinear functions are satisfactory (below we use a sine function). The reservoir output *y*(*n*) consists in a linear combination of the internal variables *x*_*i*_ with weights given by the vector *W*_*i*_, that is,


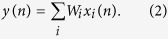


The output weights *W*_*i*_ are chosen as follows. For a given choice of parameters *α* and *β*, a “training” input series *u*(*n*) is injected in the reservoir and the internal variables are recorded. One then computes the *W*_*i*_ that minimize the Normalized Mean Square Error (*NMSE*) between the output of the reservoir *y*(*n*) and the targeted output *ŷ*(*n*)


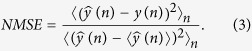


Finally, the quality of the optimized output is evaluated by means of a “test” input series *u*(*n*) injected into the reservoir while the coefficients *W*_*i*_ are kept fixed to their computed optimum values.

## Optoelectronic Reservoir Layer

**Figure 2:**
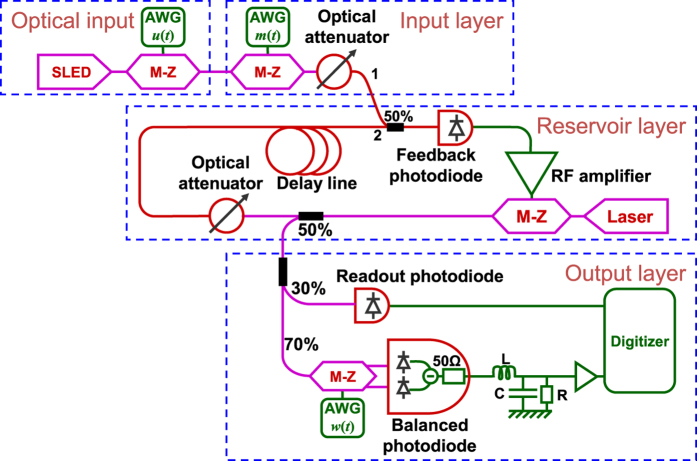
Schematic of the experimental reservoir computer. The optical input produces the signal that must be processed. The input layer multiplies the input signal by the input mask. The reservoir layer is a delay dynamical system in which a M-Z modulator acts as non-linearity. The output layer produces an analogue electric signal proportional to the desired output. Electric components are in green, optical components in red and purple, with purple used for polarization maintaining fibre components (used to avoid the use of polarisation controllers) and red for non polarisation maintaining fibre components. AWG denotes Arbitrary Waveform Generator; RF amplifier denotes Radio Frequency amplifier; R, L, C denote resistor, inductor and capacitor, respectively.

The experimental setup is depicted in Fig. 2. In the present section we describe the reservoir layer, and the next two sections are devoted to the input and readout layers. Further details on the experimental setup can be found in the Supplementary Methods and Supplementary Figure.

The reservoir layer consists of a delay line and a single nonlinear node. Similar systems have been studied previously in the general context of nonlinear dynamics, see e.g.^45^,^46^,^47^. This reservoir layer is essentially identical to the optoelectronic reservoir used in^17^, as well as in^18^,^20^,^22^,^26^,^28^,^44^. For a general overview of reservoir computing with delay dynamical systems see^48^. Such nonlinear dynamical systems with delayed feedback have intrinsically continuous time dynamics, while the fundamental equation of reservoir computing equation (1) is expressed in discrete time. Here below we will go back and forth between the continuous and the discrete time descriptions depending on the context. The correspondence between the two time scales is clearly stated when necessary.

The delay line consists of a spool of optical fibre (~1.7 km of SMF28e). The internal variables *x*_*i*_ are time multiplexed along the delay line. They are represented by the light intensity that travels along the delay line within fixed temporal windows. At the end of the fibre the optical feedback signal is added to the optical input signal injected into the cavity by the use of a 50% fibre coupler and converted to a voltage by the feedback photodiode (TTI TIA525 with a cut-off frequency of 125 MHz). The resulting signal is then amplified (ZHL-32A amplifier with a bandwidth of 50 kHz–130 MHz and a gain of 27 dB) to drive a Mach-Zehnder (M-Z) light intensity modulator (Photline MXAN-LN-10 with a bandwidth of 10 GHz). It modulates the light of a DFB laser (Covega DFB laser -SFL-1550p-NI- with a wavelength around 1550 nm). The sine response of this M-Z modulator is used as the non-linearity of the reservoir (nonlinear function *F*_*NL*_ in eq. (1)). During the experiments, the bias point of the M-Z modulator is regularly tuned to ensure a proper sine response. In other words, if no signal is applied to the RF port of the M-Z modulator, its transparency is at 50%. (Note that in some works^18^ the bias point of the M-Z is considered as a tuneable parameter which allows one to modify the nonlinear function *F*_*NL*_. Here the bias point, and hence *F*_*NL*_ is kept fixed). At the output of the M-Z modulator, 50% of the light intensity is picked up for the readout layer, the remaining 50% is attenuated by a tuneable optical attenuator (Agilent 81571A) before going into the optical delay line. This optical attenuator allows adjusting the feedback gain of the cavity (*α* coefficient in eq. (1)). The round-trip time of the cavity is *T* ≈ 8.4 μs. If we omit the constant part of the signal (that is in any case filtered out by the amplifier), the dynamics of the system without input can be approximated by


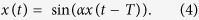


In order to carry out computation we drive the cavity with a desynchronised signal as in^17^. (Note that an alternative way to couple the internal variables to each other proposed in^15^,^18^ exploits a low-pass filter in the cavity). To this end, consider a reservoir of *N* internal variables. We define the duration *θ* of each internal variable by the relation *T *= (*N* + *k*)*θ* where we recall that *T* is the round-trip time, and *k* denotes the degree of desynchronisation. We convert the discrete time input *u*(*n*) into a continuous signal *u*(*t*) by a sample and hold procedure for duration *T’ *= *Nθ*. Thus in continuous time, the input of the reservoir computer is represented by a step signal given by *u*(*t*) = *u*(*n*) for 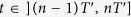.

The value of the internal variable *x*_*i*_(*n*) is given by the average value of *x*(*t*) in the interval](*i* - 1)*θ* + (*n* - 1)*T’*, *iθ* + (*n* - 1)*T’*]. The internal variable *x*_*i*_(*n*) is set to the input *u*(*n*) multiplied by the input mask value *m*_*i*_. In continuous time the input mask is represented by a periodic function *m*(*t*) of period *T’*.

The continuous time dynamics of the driven system can thus be described by


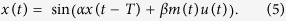


In discrete time, the variable *x*_*i*_(*n*) is connected to *x*_*i-k*_(*n*-1) if *i *> *k* or to *x*_*N + i-k*_(*n* - 2) if *i *≤ *k*. The corresponding dynamics in discrete time is thus given by


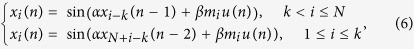


which is a particular case of equation (1).

## Analogue Input Layer

In a reservoir computer based on a delay dynamical system with a single nonlinear node, the input mask *m*_*i*_ plays a crucial role as it breaks the symmetry of the system, giving each internal variable *x*_*i*_(*n*) a different dependence on the inputs *u*(*n*). For this reason the optimisation of the input mask has been the subject of several studies^22^,^28^,^40^. In the present implementation the input mask *m*(*t*) is introduced independently of the input and is intrinsically continuous, which greatly simplifies its hardware implementation.

The optical input signal is generated as follows. A broadband source (superluminescent light emitting diode, SLED Denselight DL-CS5254A) is modulated using a Mach Zehnder (M-Z) modulator (Photline MXAN-LN-10) to generate an optical signal proportional to the input *u*(*t*) of the reservoir computer (see methods for the precompensation). This precompensated input signal is generated by an Arbitrary Waveform Generator (AWG) with a sample rate close to 200 MS/s and a resolution of 16 bits (NI PXI-5422). The intensity profile of the optical signal sent to the reservoir computer is thus given by


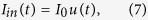


where the input has been scaled to belong to the interval *u*(*t*)∈[0,1].

The multiplication by the input mask is achieved with the same sample rate and resolution (200 MS/s and 16 bits) by another AWG (Tabor WW2074) that drives an additional M-Z modulator (Photline MXAN-LN-10). The optical signal after multiplication by the input mask has intensity


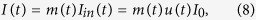


where the mask is scaled to belong to the interval *m*(*t*)∈[0,1], and for simplicity we have not written the insertion losses of the M-Z modulator.

A tuneable optical attenuator (Agilent 81571A) is used to adjust the strength of the input signal (*β* coefficient in eqs (1), (5) and (6)). The use of an incoherent light source (the SLED) avoids interferences between the signal injected into the cavity and the signal already present in the cavity (which is coherent since it comes from a laser). Therefore, at the output of the 50% fibre coupler, the feedback photodiode produces an electrical signal proportional to *αx*(*t* − *T*) + *βm*(*t*)*u*(*t*) (compare with eq. (5)).

Concerning the choice of input mask *m*(*t*), we use sinusoidal signals (as in our earlier work^44^). The simplest mask signal of this type would be a single sine at frequency *p*/*T’* with *p* integer. In this case the AWG produces the voltage


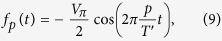


where *V*_*π*_ is the half-wave voltage of the M-Z modulator. We do not use any precompensation (as it would be incompatible with the use of a simple oscillator), which does not affect the system performance^44^. The operating point of the M-Z modulator is adjusted so that the mask is


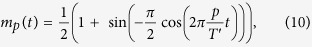


Note that the phase of the cosine in equation (9) is chosen in order to ensure that the mask vanishes at times *t = nT’* when the input *u*(*t*) has discontinuities. The signal sent into the cavity is thus a smooth function without any discontinuity and the synchronisation between the input signal and the mask is drastically simplified.

With the simple input mask equation (10), the obtained performances depend strongly on the value of *p*. For a good choice of *p*, the results are close to those we can obtain with a random input mask. However, this is true only when the output is postprocessed digitally. When the results are obtained with the experimental analogue readout layer they are significantly less good than those resulting from a random mask (we do not have a good explanation for this).

The performance is significantly improved when we use an input mask *m*_*pq*_ containing two frequencies *p*/*T’* and *q*/*T’*


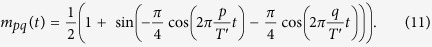


**Figure 3:**
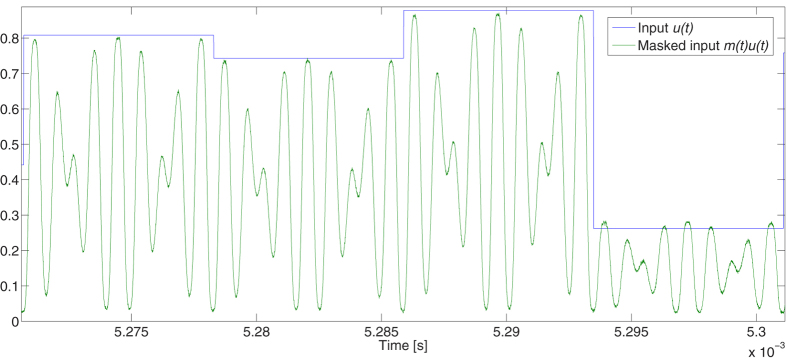
Signals injected into the reservoir layer. The blue curve is the optical input *I*_*in*_(*t*) = *I*_*0*_*u*(*t*). The green curve is a record of the masked input signal *m*_*pq*_(*t*)*I*_*in*_(*t*) with *p* = 7 and *q* = 9, as measured by a photodiode and digitizer. The vertical axis is scaled so that its maximum range is [0,1], i.e. *I*_*0*_ = 1.

The resulting optical signal fed into the cavity is again null for *t = nT’*, ensuring continuity. The mask equation (11) is the one that is used in the experiments reported here below. A trace of the masked input signal is given in Fig. 3. Note that it was necessary to scan the values of p and q to get good results.

## Analogue Output Layer

### General principle of the output layer

The readout layer is in charge of producing the output y(n) of the reservoir. It consists of two parts, the first measures the internal states x(t) of the reservoir. The second produces the output itself.

As shown in Fig. 2, 30% of the light intensity sent to the reservoir layer is detected by the readout photodiode (TTI TIA525). The resulting signal is recorded by a digitizer (NI PXI-5124) at 200 MS/s with a resolution of 12 bits and a bandwidth of 150 MHz. This signal is used during the training phase to compute the values of the internal variables *x*_*i*_(*n*) and of the readout coefficients *W*_*i*_ (following the method described below). The remaining 70% of light intensity is modulated by a dual output M-Z modulator (Photline MXDO-LN-10 with 10 GHz of bandwidth) using a signal produced by an AWG (Tabor WW2074). The two outputs of this modulator are complementary and detected by a balanced photodiode (TTI TIA527 with a cut-off frequency of 125 MHz and output impedance 50 Ω). The bias point of this modulator is regularly tuned to have a sine response. In other words, if no signal is applied on the RF port of the M-Z modulator, both outputs have a transparency of 50% and the signal at the output of the balanced photodiode is null. If a positive (negative) voltage drives the M-Z modulator, the signal at the output of the balance photodiode is positive (negative). The reason for constructing the readout layer in this way is that the internal variables are given by the optical intensity inside the reservoir, hence their values are positive. But for processing information with the reservoir computer, positive and negative readout coefficients *W*_*i*_ are required. Using a dual output M-Z modulator coupled to a balanced photodiode enables us to modulate the internal variables by coefficients that are either positive or negative. The signal from the balanced photodiode is filtered by a low-pass RLC filter whose role is to carry out an analogue summation of the weighted internal variables. The output of the low-pass filter is then amplified before being recorded by the second channel of the digitizer (NI PXI-5124). The value of the resulting signal at every instant *t *= *nT’* is the output of the reservoir *y*(*n*).

### Computation of the readout coefficients

The balanced M-Z modulator in the output layer is driven by a signal produced by an AWG. Using the method described below, one computes a continuous time weight function *w*(*t*). The signal produced by the AWG is precompensated so that the signal at the output of the balanced photodiode is proportional to *w*(*t*)*x*(*t*).

Denoting by *h*(*t*) the impulse response of the RLC filter followed by the amplifier, the signal *y*_*c*_(*t*) detected by the second channel of the digitizer can be expressed as:


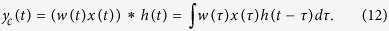


Since we use a real (causal) filter, the integration in equation (12) is done over the interval *τ*∈]−∞,*t*]. The continuous time weight function *w*(*t*) is a stepwise function of period *T’* defined by:


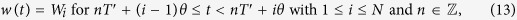


where we recall that *θ* is the duration of each internal variable. The output of the reservoir computer *y*(*n*) is a function of discrete time. It is equal to the continuous output *y*_*c*_(*t*) at time *nT’*: *y*(*n*) *= y*_*c*_(*nT’*). It can be expressed as


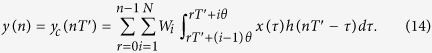


In order to calculate the readout coefficients for the analogue readout layer, new internal variables *x*_*i*_(*n*) are defined by


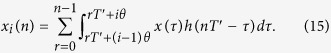


In practice, the impulse response of the readout layer has a finite length. Let *l* be an integer such that the impulse response is shorter than *lT’* (i.e. *h*(*t*) ≈ 0 for *t *> *lT’*), the sum over *r* in equations (14) and (15) can be limited to values of *r* from *n*-1-*l* to *n*-1. Note that since the impulse response lasts longer than *T’*, the current output *y*(*n*) contains contributions from the light intensity *x*(*t*) up to *l* input periods in the past, which is a small difference with respect to the traditional reservoir computer, see equation (2). In our experiment, for the channel equalization task we use *l *= 10, and for NARMA10 and the radar signal forecasting, *l *= 15.

At the beginning of the experiment, we record the step response (response to the Heaviside function) of the analogue readout layer by applying a voltage step on the dual output M-Z modulator. The derivative of the recorded signal is the impulse response *h*(*t*) of the analogue readout layer.

Note that a key point to obtain good results is to optimize the extinction of the signal when a readout weight equal to zero is applied. Because the two arms of the balanced M-Z have different insertion losses and different extinction ratios, the extinction of the signal is not obtained with a null voltage. That is why, after tuning the working point of the M-Z, we measure the voltage that corresponds to zero readout weight, and take this into account when we precompensate the readout mask.

During the training phase, we record the output *x*(*t*) of the reservoir using the readout photodiode (first channel of the digitizer). This record is then combined with the impulse response *h*(*t*) of the analogue readout layer to compute the new internal variables *x*_*i*_(*n*) (see eq. (15)). From these internal variables we compute the readout weights *W*_*i*_ using Tikhonov (Ridge) regularisation. The corresponding stepwise periodic signal *w*(*t*) is normalized with the highest absolute value of *W*_*i*_, so as to fit the maximum modulation capabilities of the analogue readout layer. The corresponding gain (the highest absolute value of *W*_*i*_) is applied on the recorded signal after acquisition of *y*_*c*_(*t*) and finally an offset correction is applied.

Note that the AWG that produces the output signal *w*(*t*) has a finite resolution, and therefore exhibits quantification noise which degrades the quality of the output *y*_*c*_(*t*). This effect is minimised if the amplitudes of the *W*_*i*_ are all comparable. This can be enforced by increasing the Ridge regularisation parameter. In the present experiments we found it useful to take a Ridge regularisation parameter 10 times larger than when we use a digital output layer. (Note that the Ridge parameter is generally used to avoid overfitting on a limited training data set. If the data set is large enough, and in the absence of the quantification noise, the Ridge parameter should be taken as small as possible in order to increase the precision of the output).

The performance of the analogue output layer is obviously dependent on the impulse response *h*(*t*), and different tasks work better with different impulse responses. In practice we first tested numerically different choices of R, L, and C, and then implemented experimentally those that provide good results. Typical values used are R in the range 1.6 kΩ–10 kΩ, C in the range 760 pF to 1.2 nF, with L = 1.8 mH.

## Results

We tested the fully analogue reservoir computer on three tasks commonly considered in the reservoir computing community, namely equalization of a nonlinear communication channel, NARMA10, and the forecast of a radar signal. We compare the results with those obtained in^17^ in which a practically identical optoelectronic reservoir computer was used, and in the case of the radar task with the all optical reservoir^19^. Both^17^ and^19^ used a similar number of internal variables, but without analogue input and output layers.

In all cases we used 47 internal variables, so *N* = 47 and *k* = 5. The two frequencies of the input mask *p*/*T’* and *q*/*T’*, are 7/*T’* and 9/*T’*. Either numerically before the experiment or during the experiment itself, the feedback gain (*α*) and the input gain (*β*) are scanned in order to find their optimal values. For each set of parameters, several datasets are used in the experiment in order to have sufficient statistics. In our experiment, a feedback gain *α* equal to 1 is obtained when the optical attenuator inside the loop is set to 9.5 dB. At this attenuation, when no input signal is applied small oscillations appear in the cavity. This corresponds to a maximum optical power received by the feedback photodiode (i.e. at maximum transparency of the M-Z modulator inside the loop) of 264.4 μW. For comparison, the optical signal received by the feedback photodiode when the input is on, and the optical attenuator in the input layer is set to 0 dB, is 1.46 mW. When the input of the reservoir belongs to the interval [0,1], the input optical attenuation to obtain a *β* coefficient of 1 is around 7.4 dB.

It is important to note that we do not carry out any time averaging on the acquired signal *y*_*c*_(*t*). For this reason the output suffers from quantification noise (see discussion below). Moreover, note that because each data set is sent to the experiment twice (once to measure *x*(*t*) and compute *w*(*t*), once to measure *y*_*c*_(*t*)), the stability of the experiment is more important than in experiments with digital postprocessing. To ensure stability, we regularly adjust the working points of all M-Z modulators.

### Nonlinear channel equalization

The aim of this task is to compensate for the distortion of a wireless communication link affected by a small nonlinearity and a memory effect. It was used previously in the reservoir computing literature, see e.g.^3^,^33^. A sequence of symbols *d*(*n*), randomly drawn from the set of values {−3, −1, 1, 3}, passes through a channel model with inter-symbols interferences (due to multi-path travels and/or band-pass filters at the channel ends) followed by a nonlinear transformation:


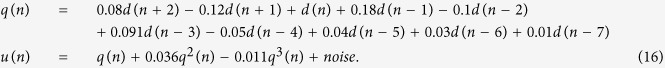


**Figure 4:**
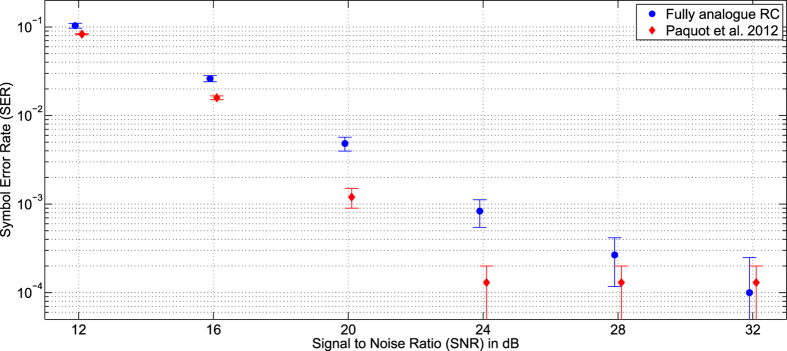
Results obtained for the equalization of the nonlinear channel for signal to noise ratios (SNR) ranging from 12–32 dB. For each SNR, the symbol error rate (SER) is given with its corresponding error bar over 5 datasets. The blue circles are the results obtained with the full analogue reservoir, and the red diamonds are the results presented in^17^ (similar optoelectronic reservoir computer, but without the analogue input and output layers).

The signal to noise ratio (SNR) is scanned from 12–32 dB using a step of 4 dB. The input of the reservoir computer is the noisy and distorted sequence *u*(*n*), while the target output is the original sequence of symbols *d*(*n*). For each SNR, the quality of the equalization is given by the symbol error rate (SER). We use 5 different datasets. For each dataset, the reservoir is trained over 3000 time steps, and then a second sequence of 6000 time steps is used to test its performances (evaluate the SER). Results are presented with their corresponding standard deviation in Fig. 4. A slight degradation is observed compared to the results obtained in^17^. The presented results are significantly better than those presented in^20^ (for instance at SNR of 32 dB, a SER of 10^−4^ compared to 10^−2^). This is due in part to the larger number of internal variables that are used (47 instead of 28), but also to a better characterisation of the output layer, and a better choice of the output filter impulse response.

**Figure 5:**
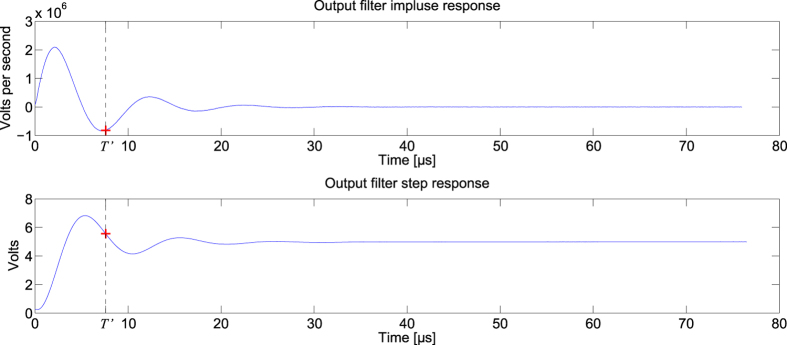
Impulse and step responses of the analogue readout layer used for the equalization of a nonlinear channel. The step response is recorded at the beginning of the experiment. Its derivative gives the impulse response of the analogue readout layer. The red cross gives the signal value at *T’* = 7.598 μs.

**Figure 6:**
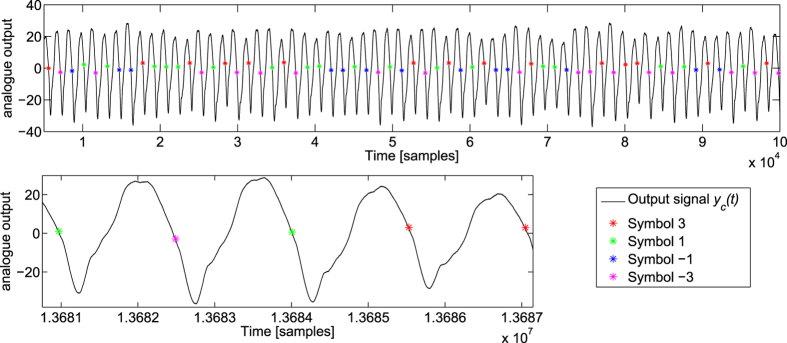
Signal at the output of the analogue readout layer for the nonlinear channel equalization task. The time is in number of samples (at 200 MS/s). The black curve is the acquired signal *y*_*c*_(*t*) with a final gain correction (multiplication by the maximum absolute value of the readout weights *W*_*i*_). The stars are the output values *y*_*c*_(*nT’*) = *y*(*n*). The different colours correspond to the different symbol values.

For this task, the feedback optical attenuator is set to 11.25 dB, the input optical attenuator is set to 5 dB, and the analogue output layer had parameters R = 1.6 kΩ, C = 1.2 nF, L = 1.8 mH. The measured impulse and step responses of the analogue readout layer are given in Fig. 5. A sample of the readout signal *y*(*t*) is given in Fig. 6. A plot of the readout weights *W*_*i*_ is given in the supplementary material.

### NARMA10

The aim of this task is to train a reservoir computer to behave like a 10^th^ order Nonlinear Auto Regressive Moving Average system (NARMA10) in which an input *u*(*n*), randomly drawn from a uniform distribution over the interval [0,0.5], is injected. The following equation defines the targeted output:


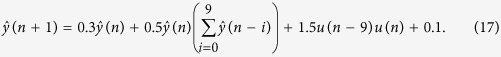


For this task, the reservoir is trained over a sequence of 1000 time steps and tested over another sequence of 1000 time steps, this process is repeated 10 times to obtain the statistics. The performance on this task is measured using the *NMSE*. This task is commonly studied in the reservoir computing community, see e.g.^33^,^49^.

**Figure 7:**
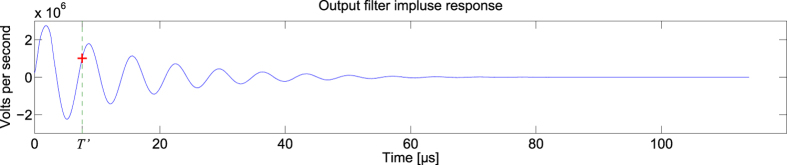
Impulse response of the analogue readout layer for NARMA10. The red cross gives the signal value at *T’* = 7.598 μs.

For this task, the feedback optical attenuator is set to 9.2 dB (i.e. slightly above the threshold for oscillations), the input optical attenuator is set to 9.5 dB, and the analogue output layer had parameters R = 10 kΩ, C = 760 pF, L = 1.8 mH. The impulse response of the analogue readout layer is given in Fig. 7.

The test *NMSE* for the all-analogue system is 0.230 ± 0.023. For the sake of comparison note that a reservoir that carries out no computation (i.e. produces a time independent output *y*(*n*) = const) has a *NMSE* = 1, the system reported in^17^ provides a *NMSE* of 0.168 ± 0.015, an ideal linear shift register (no nonlinearity in the reservoir) can reach a *NMSE* of 0.16, and using a different experimental architecture based on a coherently driven passive cavity a *NMSE* as low as 0.107 ± 0.012 was reported in^30^. Note that the all-analogue performance is slightly worse than that of the linear shift register but significantly better than a system that carries out no computation.

### Radar signal forecasting

**Figure 8:**
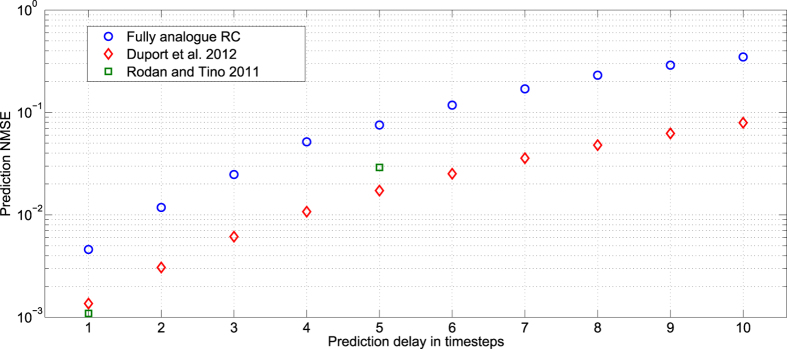
Radar signal forecasting error (*NMSE*) with respect to the number of time steps of the prediction (one to ten time steps in the future). The blue circles are the results obtained with the fully analogue reservoir, the red diamonds are the results published in^19^ obtained with an all optical reservoir computer with similar number of internal variables, but without the analogue input and output layers. The green squares are the numerical results of^33^.

This task consists in predicting a radar signal one to ten time steps in the future from a radar signal backscattered from the ocean surface (data collected by the McMaster University IPIX radar). The quality of the forecasting is evaluated by computing the *NMSE* between the predicted signal and the actual data one to ten time steps in the future. The experiment uses a single recorded radar signal under low sea state conditions, corresponding to an average wave height of 0.8 meters (max 1.3 m). The recorded signal has two dimensions, corresponding to the in-phase and in-quadrature outputs (respectively, *I* and *Q*) of the radar demodulator. Therefore for each dataset, the in-phase and in-quadrature signals are successively processed (predicted) by the experiment. The training and test sequences contain 1000 inputs each. This task has been previously used to evaluate the performance of reservoir computers, see e.g.^33^,^50^. The results are presented in Fig. 8.

**Figure 9:**
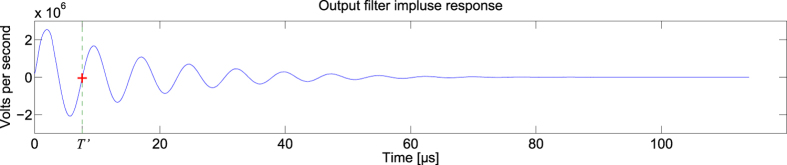
Impulse response of the analogue readout layer used for the radar signal forecasting. The red cross gives the signal value at *T’* = 7.598 μs.

For this task, the feedback optical attenuator is set to 9.9 dB, the input optical attenuator varied between 7 and 10 dB, and the analogue output layer had parameters R = 10 kΩ, C = 810 pF, L = 1.8 mH. The impulse response of the analogue readout layer is given in Fig. 9.

## Discussion

The novel feature of the experimental reservoir computer presented here is the simultaneous inclusion of analogue input and output layers. The interest of this configuration is that it represents the necessary step towards develop standalone reservoir computers for future complex and high-bandwidth applications. The only role of the external computer in our experiment is to compute the output weight function *w*(*t*).

Concerning the analogue input layer, we proposed the use of sinusoidal functions as input mask, as these will be simple to generate in future hardware implementations. Upon using the sum of two sines as input mask, we did not observe significant degradation of performance compared to using the standard random step function.

The present analogue input layer is well suited to scalar input signals. However there are many problems in which reservoir computers are used to process a vector input signal *u*_*i*_(*n*). This is for instance the case in the radar task of Sec. 6.3 in which both the *I* and *Q* channels may be used as input (we used here either *I* or *Q*), and in many speech recognition tasks where the input consists of several auditory channels. We shall address this issue in forthcoming works.

Concerning the analogue output layer whose aim is to produce a linear combination of the internal states that yields the desired output, the key difficulty is the accuracy needed in the summation that involves a large number of adjustable factors (the output weights).

The results presented here are obtained without any temporal averaging of the recorded signal, which makes them sensitive to quantification noise. This is important in our case since the total range of the output signal *y*_*c*_(*t*) is much larger than the range of the outputs *y*_*c*_(*nT’*) = *y*(*n*), see Fig. 6. In the case of the channel equalisation task, which essentially constitutes a classification task, quantification noise is not such a problem since a signal that is correctly classified will in general continue to be so if a small amount of noise is added. But in the case of NARMA10 and the radar task, we measure the performance by how close the output is to the desired output using the *NMSE*. Quantification noise then directly affects the performances. (Note that the effects of quantification noise and methods to counteract it have been studied previously in the context of reservoir computing in^22^,^26^).

Quantification noise also affects the readout mask *w*(*t*). For this reason the Ridge regularisation parameter was optimised in order to minimise the range of *w*(*t*), as discussed in section 5.2.

We emphasize that for different tasks, different output filters were used (values of the constants R, L and C). We do not have a complete explanation of why the optimal filters are different for each task. In our previous experiment^20^, we used a simpler RC filter. This filter typically has a long impulse response, but on the other hand the resulting signal is much smaller, which leads to an increase of the output quantification noise. In the present work, we used a second order RLC filter that also exhibits a long impulse response, but keeps a larger signal range.

In summary, we proposed in this work the first study of a fully analogue reservoir computer. At stake is the development of future analogue computers dedicated to complex and high-bandwidth signal-processing tasks. Due to the added complexity of our experiment, some degradation of performance is naturally observed compared to previous experiments in which the input and output layers were implemented digitally through digital pre and post processing. However, the present experiment can be considered as a proof of principle that suggests the feasibility of fully standalone reservoir computers. In this sense our work can also be seen as an important step towards the development of novel applications in which reservoir computers are cascaded or looped on themselves. As emphasized in the above discussion, many technical problems remain to be solved. For instance, to circumvent some of the difficulties related to the use of fast-electronics we are currently addressing the possibility of implementing an all-optical output layer.

## Additional Information

**How to cite this article**: Duport, F. *et al*. Fully analogue photonic reservoir computer. *Sci. Rep*. **6**, 22381; doi: 10.1038/srep22381 (2016).

## Supplementary Material

Supplementary Information

## Figures and Tables

**Figure 1:**
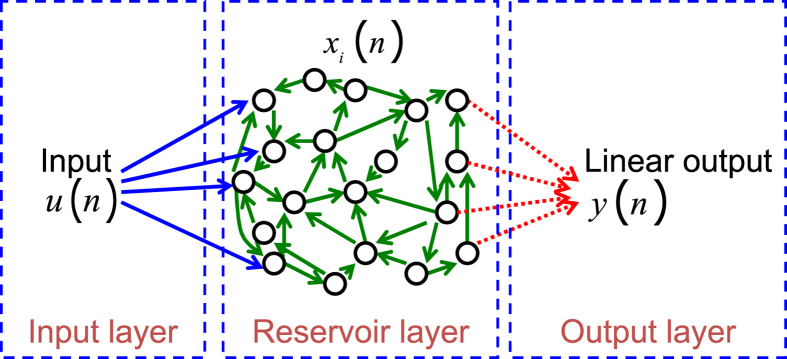
Schematic of a reservoir computer. The input *u*(*n*) is sent to the reservoir layer by the input layer. The reservoir layer is a nonlinear recurrent dynamical system whose internal variables are denoted *x*_*i*_(*n*). The output *y*(*n*), produced in the output layer, is a linear combination of the internal variables. Only the linear output layer is adjusted during the training phase.
