# Supplementary material for: Fully analogue photonic reservoir computer
Source: Sci Rep. 2016 Mar 3;6:22381. doi: 10.1038/srep22381 (PMC4776108; doi:10.1038/srep22381)
Supplement: Supplementary Information [file srep22381-s1.pdf]

Fully analogue photonic reservoir computer

François Duport[1], Anteo Smerieri[1], Akram Akrout[2], Marc Haelterman[1] and Serge Massar*[2]

[1] OPERA-Photonic, CP 194/5, Université Libre de Bruxelles (U.L.B.),
Avenue Adolphe Buyl 87, 1050 Brussels, Belgium

[2] Laboratoire d'Information Quantique, CP 225, Université Libre de Bruxelles (U.L.B.),
Boulevard du Triomphe, 1050 Brussels, Belgium

• Corresponding Author : smassar@ulb.ac.be

Supplementary Methods: Tips and tricks for building a fully analogue photonic reservoir computer

1. Introduction

The purpose of this supplementary material is to give indications on how to reproduce our experiment. If the basic principle of photonic reservoir computing based on a delay line oscillator is rather simple, the challenges lie in the experimental setup and the test bench used to drive the experiment. In the present supplementary material we give indications for building such an experiment. Firstly we discuss the design of the experiment, including the choice of components and their most important parameters. Secondly we discuss how to tune the test bench. Finally we discuss the use of the Mach-Zehnder modulators, including the precompensation of the signal and the tuning of their operating point.

2. Experimental setup

Our experimental setup is depicted in figure 2 of the main text. As already explained, the core of this architecture (the reservoir layer), is a delay line optoelectronic oscillator. An optical input signal is injected in this oscillator to drive the experiment, and a part of the light intensity is extracted from the oscillator to record signals and to construct the analogue output. The first things to consider when building such experiments are the optical and electrical budget inside the oscillator and in the output layer, as well as how to choose the parameters of the optical and electrical components. The basic idea here is to try to keep the signal to noise ratio as high as possible, while keeping all the components (except the MZ in the oscillator) in a linear regime.

When using such an oscillator as a physical reservoir computer, the feedback gain of the oscillator is close to 0dB. Note that for NARMA10 task this gain is +0.3dB, while for the other tasks it is slightly below 0dB. All the photodiodes used in our experiment (TTI TIA525 and TTI TIA527) exhibit a linear behaviour when the optical power is below 1mW. The M-Z modulator used inside the oscillator has a half-wave voltage (V_π) of 6.5V. Denoting by P_{max} the maximum optical power available on the photodiode, by G_{RF} the gain of the RF amplifier, by G_{PD} the gain of the photodiode, the optical power P_{PD} received by the photodiode and the voltage $V(t)$ which drives the M-Z modulator are given by

$$\begin{aligned} P_{PD}(t) &= P_{\max}\frac{1}{2}\left(1+\sin\left(\frac{\pi}{V_\pi}V(t-T)\right)\right), \\ V(t) &= G_{RF}G_{PD}P_{PD}(t)\ . \end{aligned} \tag{1}$$

At the edge of stability (feedback gain of the oscillator close to 0dB), only small oscillations occur. Therefore, if $v(t)$ are the small variations of the voltage, the optical power received by the photodiode is

$$P_{PD}(t) = P_{\max} \frac{1}{2} \frac{\pi}{V_\pi} v(t-T), \qquad (2)$$

and the cumulated voltage gains G_{PD} of the feedback photodiode and G_{RF} of the RF amplifier are

$$G_{RF} G_{PD} = \frac{V_\pi}{\pi} \frac{2}{P_{\max}}. \qquad (3)$$

Hence, if the maximum optical power available on the photodiode, P_{max}, is 1mW, then ideally the cumulated voltage gains $G_{RF}G_{PD}$ should be 4138 V/W.

In our experiment the gain of the feedback photodiode is 1400 V/W and the gain of the RF amplifier is 27dB, corresponding to a voltage gain of 22.36. The cumulated gain of the photodiode and RF amplifier is therefore approximately 15650V/W (taking into account the 50Ω impedance of the photodiode and RF amplifier). This gain is larger than the optimum calculated above, but this does not degrade significantly the performances of the experiment, as it is compensated by increasing the attenuation within the optical cavity.

Moreover, when considering the optical signal injected in the reservoir layer, the power received by the feedback photodiode is the sum of the optical input and feedback signals. So a larger cumulated gain of the feedback photodiode and RF amplifier (compensated by an optical attenuation within the cavity) enables the experiment to run at the edge of stability with a strong driving (injected) signal.

Let us consider now the optical signal injected in the optoelectronic oscillator (the optical input and input layer in figure 2). The only rule to follow is to keep the feedback photodiode within its linear regime. In other words, taking into account the insertion losses of the two M-Z modulators, the optical attenuator and the 50% optical coupler, the optical power coming from the input layer should not exceed much 1mW. In our experiment, when setting the two M-Z modulators at maximum transparency and the optical attenuator at 0dB, we measured a maximum power coming from the input layer of 1,46mW.

For the readout layer, the first thing to consider is the position inside the optoelectronic oscillator where the signal is picked up to construct the output signal. In our experiment, we chose to couple out part of the optical signal just after the M-Z modulator and before the optical attenuator that defines the feedback gain of the reservoir. In this configuration, the extracted signal is always bounded between 0 and a constant maximum optical power, independently of the strength of the optical input and feedback signals. Inside the output layer, the 30% - 70% ratio of the optical coupler is chosen so that the maximum power on the readout photodiode is comparable to the maximum power on each input of the balanced photodiode. The coupling ratio of the optical coupler which extracts the signal from the reservoir layer and the optical power delivered by the laser inside the reservoir layer are adjusted to have a maximum optical power on the readout and balanced photodiodes around 1mW.

When measuring the impulse response of the analogue readout, the M-Z modulator inside the reservoir layer is set to its maximum transparency. This corresponds to the maximum possible value of the internal variables inside the reservoir layer. The corresponding voltage at the output of the photodiode which records the optical intensity inside the reservoir layer is measured, this value is used to normalise the internal variables during the experiment. The impulse response of the analogue readout is derived from the record of the response to a step voltage from $-V_\pi/2$ to $V_\pi/2$ applied on the balanced M-Z modulator. These procedures enable us to precisely predict how the weights W_i are related to the amplitude of the analogue output $y_c(t)$.

3. TEST BENCH

The test bench which drives our experiment is composed of two arbitrary waveform generators (AWG NI-PXI5422 and Tabor WW2074) and one digitizer (NI-PXI5124). All these devices are synchronized by sharing the 10MHz reference clock of the NI-PXI5422. The signals for the input mask and for the readout mask are periodic. For a good synchronisation of these signals with the input signal

and the reservoir layer, the sampling rate of all the AWGs is chosen such that the number of samples within one internal variable duration θ is constant (equal to 32 in our experiment which corresponds to a sampling rate of 197.9MS/s). Moreover, the sampling frequency is chosen to be as close as possible to the maximum achievable 200MS/s so as to achieve the best signal generation. The digitizer (NI PXI-5124) does not enable the same flexibility in the choice of the sampling frequency. Indeed the sampling frequency of the digitizer can only be 200MS/s divided by an integer number (200MS/s, 100MS/s, 66MS/s etc.). In our experiment the sampling frequency of the digitizer is always 200MS/s.

4. M-Z MANAGEMENT

The M-Z modulators in our experiment are regularly tuned such that they exhibit a sine response to the applied voltage. Here we explain how we precompensate the signal which drives the MZ modulators inside the optical input layer and inside the readout layer (see figure 2 in the main text) so as to obtain a linear response.

Let's consider the light intensity I_{in} at the input of a M-Z modulator, with the insertion loss ρ and half wave voltage V_π. The light intensity I_{out} at the output of the modulator as a function of the driving voltage $v(t)$ is given by:

$$I_{out}(t) = \rho I_{in}(t)\frac{1}{2}\left(1+\sin\left(\frac{\pi}{V_\pi}v(t)\right)\right). \qquad (4)$$

The goal of the precompensation is to obtain at the output of the MZ modulator a light intensity proportional to the input light intensity multiplied by the signal $f(t)$. This is obtained by taking $v(t)$ to be equal to

$$v(t) = \frac{V_\pi}{2}\frac{2}{\pi}\operatorname{Arcsin}\left(f(t)\right) \qquad (5)$$

where we assume that the signal $f(t)$ belongs to the interval [-1 1]. Therefore the signal $g(t)$

$$g(t) = \frac{2}{\pi}\operatorname{Arcsin}\left(f(t)\right) \qquad (6)$$

should be loaded in the AWG and generated with an amplitude of $V_\pi/2$.

In the case of a dual output MZ modulator, the light intensities I_{out1} and I_{out2} at the two outputs of the modulator are similarly given by:

$$\begin{aligned} I_{out1}(t) &= \rho I_{in}(t)\frac{1}{2}\left(1+\sin\left(\frac{\pi}{V_\pi}\frac{V_\pi}{2}g(t)\right)\right) = \rho I_{in}(t)\frac{1}{2}\left(1+f(t)\right) \\ I_{out2}(t) &= \rho I_{in}(t)\frac{1}{2}\left(1-\sin\left(\frac{\pi}{V_\pi}\frac{V_\pi}{2}g(t)\right)\right) = \rho I_{in}(t)\frac{1}{2}\left(1-f(t)\right) \end{aligned} . \qquad (7)$$

Hence when detecting these two outputs with a balanced photodiode, the resulting signal should be proportional to the light intensity at the input of the modulator multiplied by the signal $f(t)$.

However the balanced M-Z modulator and balanced photodiode have some imperfections, namely different insertion losses and on/off ratios for the two outputs of the modulator, and the difference in responsivity of each photodiode. For these reasons a null voltage on the RF port of the M-Z modulator does not give, at the output of the readout layer, full extinction of the optical input. This effect, if not taken into account, degrades the performance of the output layer. To compensate for it, we measure the small offset needed to obtain a full extinction of the signal at the output of the reservoir layer. This offset is applied as a small correction to the signal driving the output M-Z modulator.

The following figure gives the response after the balanced photodiode with and without precompensation when a ramp of 47 weights W_i ranging from -1 to 1 is use for the analogue readout mask.

Supplementary Figure1: Precompensation of the dual output MZ modulator.

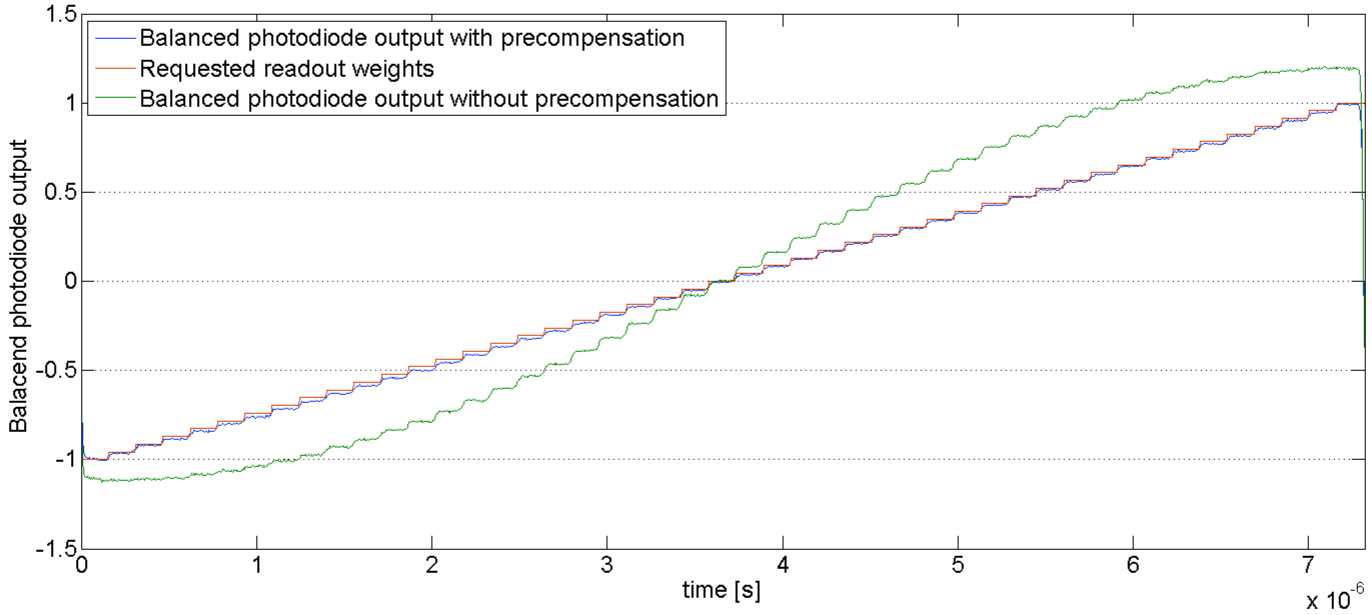

Precompensation of the dual output MZ modulator. The signal *f*(*t*) used for this test is a stepwise function of 47 values from -1 to 1 over the period *T'* (red curve). The green record is the output of the balanced photodiode when no precompensation is applied on *f*(*t*). The blue curve is normalized output of the balanced photodiode when the precompensation is used.

Supplementary Figure2: Readout coefficients for channel equalization

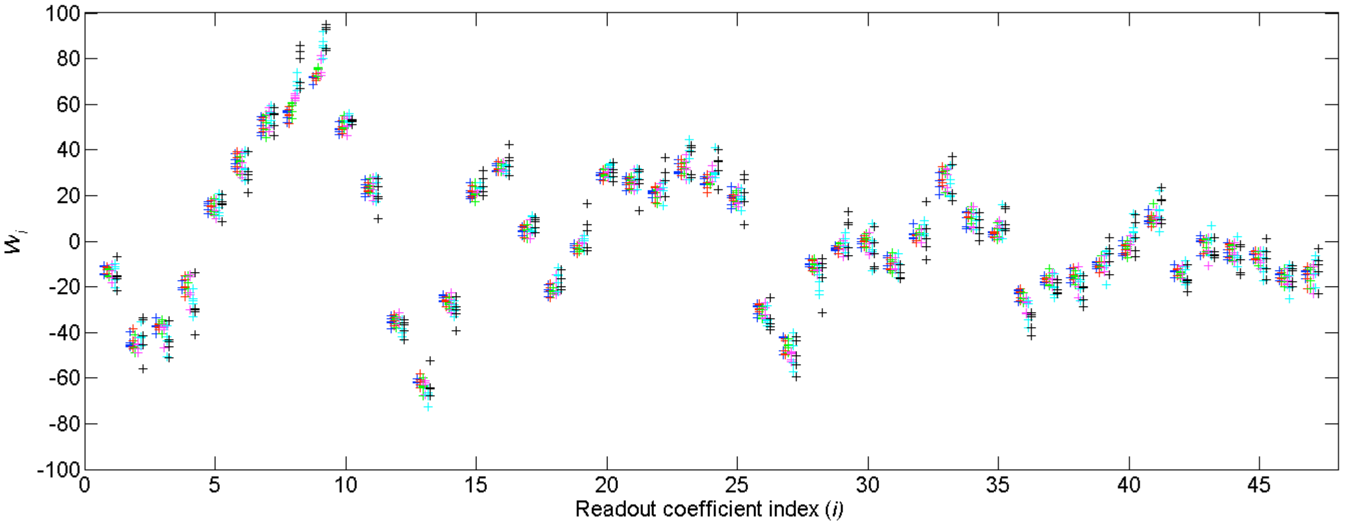

Readout coefficients for nonlinear channel equalization. The readout coefficients W_i are given for the six investigated SNRs: 32dB SNR is in blue, 28dB in red, 24dB in green, 20dB in magenta, 16dB in cyan, and 12dB in black. The output signal is taken at the end of the 47th internal variable. Vertical scale is arbitrary. Recall that for each investigated SNR, five independent datasets were used (see main text for details). The readout coefficients were computed independently for each data set. For each SNR we have plotted these five sets of readout coefficients. Thus for each index *i* we have plotted 30 values W_i (5 datasets per SNR and 6 SNRs). One sees from the figure that the values W_i for a given index *i* are all very similar. This is not unexpected since the tasks corresponding to different SNRs are very similar.
